# The cost of incentive compatibility in auction‐based mechanisms for carrier collaboration

**DOI:** 10.1002/net.21828

**Published:** 2018-06-27

**Authors:** Margaretha Gansterer, Richard F. Hartl, Rudolf Vetschera

**Affiliations:** ^1^ Faculty of Business, Economics and Statistics, Department of Business Administration University of Vienna Oskar‐Morgenstern‐Platz 1 Vienna 1090 Austria

**Keywords:** auctions, collaboration, game theory, incentive compatibility, logistics, transportation

## Abstract

Collaboration has been one of the important trends in vehicle routing. A typical mechanism to enable carrier collaboration is to use combinatorial auctions, where requests are not traded individually but are combined into bundles. Previous literature on carrier collaboration has focused on issues such as bundle formation or winner determination, typically assuming truthfulness of all agents and absence of any strategic behavior. This article considers the interdependencies and problems that arise from bidders acting as buyers and sellers of requests at the same time. From standard auction theory, desirable properties of exchange mechanisms are identified as efficiency, incentive compatibility, individual rationality, and budget balance. It is shown that these desirable properties cannot be fulfilled at the same time. In particular, the properties efficiency and incentive compatibility induce that budget balance is violated, that is, an outside subsidy is required. We propose two incentive compatible exchange mechanisms. One is more closely related to the classical VCG approach, while the other one uses a more complicated concept for computing payments to participants. A numerical study investigates how frequently desired properties are violated. We show that both mechanisms can be acceptable in practical situations, but none of them can satisfy all desired properties.

## 1 INTRODUCTION

Collaborative relationships have been recently identified by Speranza 57 to be one of the big trends in transportation. Statistics show that in Europe approximately 90% of freight travels on road, where the percentage of empty trucks contributing to traffic, pollution, and accidents is between 15% and 30%. The authors argue that the average load of a truck is much lower than its capacity and particularly low in city distribution, creating many opportunities to improve capacity utilization. Collaboration among carriers may reduce inefficiencies in operations, increase capacity utilization, and thus generate economic benefits for the participants involved as well as for the society 6, 57.

**Figure 1:**
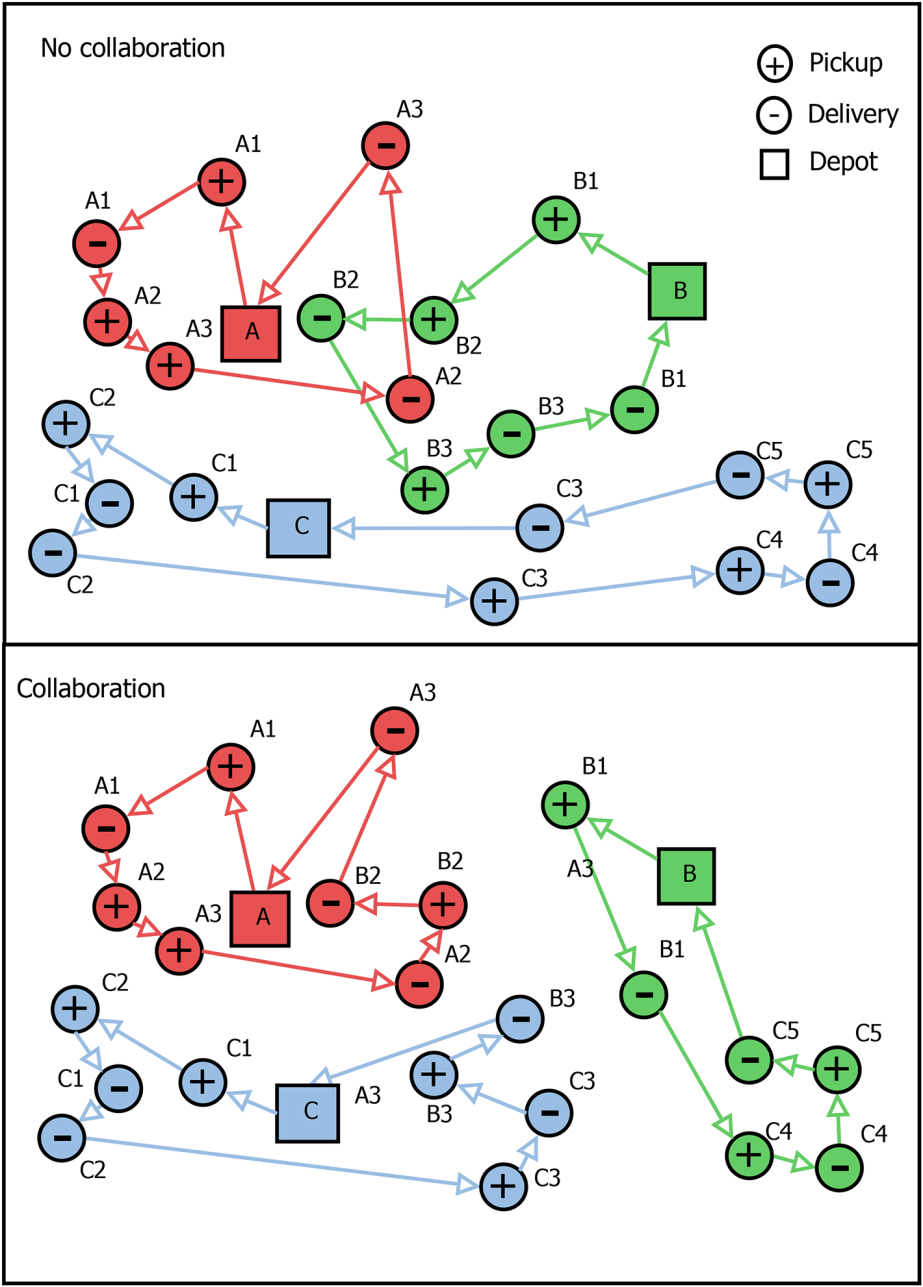
Example for non‐collaborative and collaborative vehicle routes of three LTL carriers with pickup and delivery requests 28

In horizontal logistics collaboration, several companies pool their transportation requests in order to execute them more efficiently 17. The results of these initiatives are impressive: double‐digit efficiency improvements of up to 30% have been reported 28, 59. Figure 1 shows an example of noncollaborative and of collaborative vehicle routes of LTL carriers.

Horizontal collaboration in logistics requires the establishment of an exchange mechanism for requests, which is frequently implemented via an auction‐based mechanism. In combinatorial auctions requests are not traded individually, but combined into packages (or bundles). The main reason for bundling is that the value of a request to a carrier often depends on the availability of other requests that a carrier can fulfill. A carrier receives the full bundle if the bidding price is accepted. If the bid is rejected, none of the items contained in the package is acquired. This eliminates the risk of only obtaining a subset of requests which does not fit into the current request portfolio (e.g., because the requests are geographically distant from the other requests of the carrier). Hence, combinatorial auctions are generally seen as a very effective mechanism to allocate transportation requests (e.g., [2, 52, and 56]).

Auctions are not the only mechanism that could be used to implement an exchange of requests among carries. In particular, the term logistics collaboration suggests to consider also mechanisms of collaborative decision making, in which a group of participants jointly decide on a solution (in that case, an allocation of requests to carriers) that is optimal from the perspective of the entire group. Approaches to group decision making frequently assume that group members share all the relevant information required to solve the problem at hand fully, openly and truthfully, a condition often referred to as FOTE (Fully Open Truthful Exchange, 51). However, in our view, this assumption is particularly problematic for the problem we are studying. Although carriers will want to exchange several requests, there will also be requests that carriers want to retain, either because it is obvious that they are in a good position to fulfill these requests, or for other reasons like keeping an important, strategic customer. Still, finding an optimal allocation would require them to share information beyond the requests they intend to exchange (e.g., total capacity, and information about requests they do not share). Such additional information beyond the actual group decision problem, and the disincentives for sharing it, are not considered in models of group decision making.

To avoid revealing this information, carriers might even have incentives to distort information about the requests they actually intend to trade. Literature on group decision models frequently argues that the complexity of the problem makes it difficult to successfully manipulate such a process and players will therefore provide correct information on the actual group decision problem. However, it can be shown 61 that even simple manipulation attempts, although they do not provide the optimal response of a player to the other's strategies, can be quite beneficial for a manipulating actor. Therefore this article, in accordance with much of the literature on carrier collaboration 28 considers auctions as an adequate mechanism in which carriers have to provide only a limited amount of information.

In our computational study, we focus on less‐than‐truckload (LTL) carriers being in charge of pickup and delivery requests. The carriers are willing to exchange their requests with their competitors, aiming for an increase in efficiency and sustainability. They do so using combinatorial auction‐based mechanisms, where we assume that carries determine their bids based on their marginal costs for the traded requests. The winning carrier serves the request and is compensated by the price which has been a priori negotiated with the customer. Requests that are offered for auction, can be exchanged without contractual conflicts. Our mechanism offers the possibility of keeping private requests, that is, orders that carriers are not willing to share. Details on the decision problem faced by the participants are given in Section 6.

Although many auction‐based mechanisms for carrier collaboration have been proposed in literature, this problem has several characteristic features that distinguish it from usual settings in which auctions are employed. First, carriers may at the same time and in the same auction act as buyers and sellers. Second, even the designation as buyers and sellers is not entirely clear. On the one hand, one can consider a request to be transferred to another carrier as a “good,” and therefore designate the carrier who passes on a request as the “seller” of that request, and the carrier who acquires the request as “buyer.” On the other hand, one might consider the execution of a request as a service that is offered (and thus “sold”) by the carrier taking over the request, and “bought” by the carrier who outsourced the request. In this article, we will follow the second interpretation and designate the carrier who passes on (outsources) a request as the buyer and carriers who offer to execute (insource) the request as sellers of the request.

Several decisions have to be made in the process of re‐allocating transportation requests via combinatorial auctions 12:

1. Which requests are to be transferred from one carrier to another carrier? (request selection)
2. How are requests combined into bundles of requests? (Bundling)
3. How are bundles evaluated by carriers? (Bidding)
4. How are bundles allocated to carriers? (Winner determination)
5. How are payments from or to carriers determined? (Payment determination)

Standard auction models characterize auctions by two functions; one represents the probability of winning the auction, which describes the winner determination, and a payment function, which describes the payment determination. Decisions 4 and 5 are therefore covered by standard auction theory. Many models proposed in literature for collaborative logistics assume that request selection is performed by carriers in a first step preceding the entire mechanism. However, there are important interdependencies between that step and the following steps, since the costs that a carrier incurs by executing a request him‐ or herself depend on the outcome of the entire process. These interdependencies also imply that in our view, bundling of requests has to consider requests that are insourced as well as requests that are outsourced by a carrier; separating these two operations ignores interdependencies between requests. We therefore do not address these two decisions as separate steps of the process, but consider them to be part of a comprehensive framework. As far as bidding is concerned, two cases can be distinguished: If the mechanism leaves room for strategic behavior, then bidding will involve strategic considerations by carriers, otherwise, self‐interested carriers will bid their true evaluations. Which of the two situations applies depends on how the mechanism for steps 4 and in particular 5 is designed.

In auction theory, auctions that involve multiple buyers and multiple sellers are known as double auctions 67. Typical examples for double auctions are stock markets, where multiple buyers and sellers trade shares. However, the present setting is different from the usual setting of double auctions, since each request is unique. Thus, only one carrier acts as buyer for each request, and only one unit of each type of request can be traded. This creates a monopoly situation for buyers. In contrast, in a stock exchange, many identical shares in the same firm are typically traded by multiple buyers and sellers. Although there is only one request of each type, that request may exhibit positive or negative synergies with other requests both on the buyer and the seller side. A buyer's marginal costs for performing a given request herself (and thus the maximum cost at which the request should be outsourced) may depend both on whether other requests are outsourced or not, as well as on the requests insourced and executed for other carriers. These complex interactions make the problem particularly difficult and present specific challenges for the design of an adequate mechanism that we will explore in this article.

There are several desirable properties that a mechanism for allocating goods (and in our case, transportation requests) should fulfill. The first is efficiency (EF) of the resulting allocation. In a standard auction setting, an EF allocation is often defined as an allocation that assigns each good to the agent who values it highest 42, 67. However, in a combinatorial setting with synergies, the value that a carrier assigns to a request depends on the other requests the carrier must fulfill, so a unique “value” of a request does not exist. We therefore define an allocation as EF if no further gains from trade are possible 67, or equivalently, if the allocation maximizes value creation from exchange.

The second property is incentive compatibility (IC). We consider a direct mechanism, in which each agent indicates his or her valuation of a bundle of requests to the mechanism, and the mechanism then performs the allocation and determines payments. A mechanism is IC if stating the true valuation (which exists for a bundle, although not for a single request) is the optimal strategy for each agent. According to the revelation principle 45, it is always possible for any mechanism to find an equivalent IC mechanism, that generates the same equilibrium allocation. Therefore, IC does not restrict the possibility to find an adequate mechanism for a problem, but still it is a property that a meaningful mechanism needs to fulfill.

Another important property is individual rationality (IR). No carrier should be worse off participating in the exchange than when not participating. This is obviously a requirement for a feasible mechanism. If carriers had incentives to abstain from the exchange, potentially value‐creating trades would not occur.

Finally, since we consider the mechanism to be a collaborative effort that is jointly organized by carriers, we have to take budget balance (BB) into account. In most auction settings, BB refers to the requirement that the auctioneer does not incur a loss. However, if the auctioneer is an entity that is jointly created and operated by the carriers, the opposite is also not desirable. If the auctioneer makes a surplus, that surplus needs to be allocated among the participating carriers and this could endanger IC (if carriers, who are also bidders in an auction anticipate that deviating from reporting their true valuations, although harming them as bidders in the auction, might increase their share in the auctioneer's payoff).

Existing theory already indicates that these requirements cannot all be fulfilled simultaneously by one exchange mechanism. For a bilateral exchange between one buyer and one seller for one good, Myerson and Satterthwaite 46 have shown that a mechanism that is IC for both sides and leads to an EF allocation requires an outside subsidy, that is, is not BB. Similar results also hold for double sided auctions 67. In this article, we will therefore explore possible trade‐offs between these requirements. In particular, we will show that IC and IR in that specific setting will lead to significant deviations from BB, which then, once the requirement to finance the resulting deficit of the auctioneer is taken into account, has repercussions on the former criteria.

We propose two exchange mechanisms in order to examine the trade‐off among the proposed properties. One is more closely related to the classical VCG approach, while the other one, called team bidder, uses a more complicated concept for computing payments to participants. In both cases, the resulting deficit of the auctioneer needs to be charged to participants. We show that this might ex post lead to violations of IR. A numerical study based on the above theoretical findings investigates how frequently the desired properties are violated ex ante (before compensating the auctioneer) and ex post (after compensating the auctioneer). We show that both mechanisms can be acceptable in practical situations, but none of them can satisfy all desired properties.

The remainder of the article is structured as follows: In Section 2, we provide a literature review on the use of (combinatorial) auctions to organize collaboration between carriers. In Section 3, we begin our analysis by considering a comparatively simple case of exchanging single requests between carriers, which ignores interdependencies between requests. In Section 4, we analyze another simplified setting, and consider an exchange mechanism for bundles of requests, which does not take into account IC for buyers. Then, in Section 5, we consider both aspects of the problem and analyze a model for combinatorial, double‐sided exchange. Section 6 presents a computational study that compares different mechanisms for the latter case, and Section 7 concludes the article by discussing its main results, limitations, and future research.

## 2 LITERATURE REVIEW

The first studies to systematically assess the potentials of collaborative vehicle routing were presented by Krajewska and Kopfer 35 and Cruijssen et al. 17. Since then, this topic has been extensively researched. Related literature reviews are presented by Cruijssen et al. 18, Verdonck et al. 60, Guajardo and Rönnqvist 33, and Gansterer and Hartl 28. Recently, the focus has been extended from pure cost minimization to ecological goals, like reduced road congestion, noise pollution, and emissions of harmful substances 44, 50, 53.

If collaborative decisions are made by a central authority having full information, this is referred to as centralized collaborative planning (e.g.,^3^, ^14^, ^21^, ^25^, ^29^, ^36^, ^37^, ^41^, and 65). If players are not willing to give full information to a central planner, decentralized mechanisms are needed. In this case, collaborators might cooperate individually or be supported by a central authority.

Research on decentralized planning considers both auction‐based and nonauction‐based mechanisms. In our study, we are focusing on auction‐based exchange mechanisms. However, readers interested in non‐auction based systems are referred to, for example,^4^, ^19^, ^23^, ^24^, ^34^, ^47^, ^58^, ^63^, ^64^, ^65^.

Auctions are generally seen as an effective mechanism to exchange transportation requests (e.g.,^2^, ^52^, ^56^). They are supposed to be more complex than their conventional (i.e., nonauction‐based) counterparts. However, auctions have more potential, since the trading mechanism can be used to indirectly share information of collaborators' preferences. The first auction‐based exchange mechanism for collaborating carriers is presented by Krajewska and Kopfer 35.

In combinatorial auctions, requests are not traded individually but in packages 1, 49. These mechanisms are of particular relevance in the pickup and delivery market, where shipments from several different customers can be transported on a single vehicle 7, 31, 40.

A combinatorial exchange mechanism exploiting synergies for transportation routes of various carriers is presented by Schwind et al. 55. Ackermann et al. 2 discuss goals for a combinatorial request exchange in freight logistics aiming at improving usefulness in a practical environment of LTL carriers. A combinatorial clock‐proxy exchange for carrier collaboration is presented by Chen 15.

The decision, which requests to offer for trading is investigated by Gansterer and Hartl 26 and Schopka and Kopfer 54. A critical point is the dimensioning of the auction pool. From a practical point of view, trading all possible packages (bundles) of requests is not manageable, since the number of bundles grows exponentially with the number of requests that are in the pool. To overcome this problem, Li et al. 39 do not allow the carriers to submit more than one request to the auction pool. In the multi‐agent framework presented by 20, there is only one request traded per auction round. However, limiting the number of offered items, obviously decreases the probability to find good solutions. Gansterer and Hartl 27 show how, with only a small loss in solution quality, the set of offered bundles can be efficiently reduced to a relatively small subset of attractive ones.

Complexity can be reduced by using multi‐round auctions (e.g., Ref. 22). These are generally intended to offer subsets of the traded items in multiple rounds. Previously gained information can be used to compose the setting for the next round. Consequently, the bidders are never faced with the full complexity of the auction pool.

None of the papers mentioned above have looked at the game theoretic aspect, or more precisely, whether the properties EF, IC, IR, and BB are satisfied. A notable exception is Xu et al. 68, who present truthful, BB bundle double auctions for full truckload carrier collaboration. However, they assume that a carrier cannot both be the buyer and seller during one auction round. Also, Lai et al. 38 assume that carriers can only be either seller or buyer at the same time. They further assume that each buyer can only be assigned with at most one request. Babaioff and Walsh 9 investigate the trade‐off among desirable properties in supply chain formation auctions. They assume that bidders are buyers and sellers at the same time, but not in the same market.

To the best of our knowledge, we are the first to explore the problems arising in exchange mechanisms, where carriers can be sellers and buyers at the same time in the same market. Since under this assumption, all desirable properties (IC, EF, BB, IR) cannot be fulfilled simultaneously, we study the possible trade‐offs between these properties. Specifically, we show that in the specific setting of exchanging interdependent transport requests, BB poses a particular problem and requires weakening the other requirements.

## 3 SINGLE REQUEST MECHANISM

In the first model, we consider the exchange of single requests and thereby ignore possible (positive or negative) synergies between requests being exchanged. Note that this does not mean that synergies between requests that are traded, and other requests that stay in the original carrier's portfolio of requests are not taken into account. The main consequence of this assumption is that all parties involved (buyer and sellers) can assign unique marginal costs to each request being traded. This assumption could be realistic if only few requests are traded, so that giving up or receiving some requests will not change the optimal routing significantly (e.g., additional requests can simply be inserted into existing routes without changing the rest of the route or the assignment of other requests to routes). Apart from this possible application, analyzing this case will also help to explain the effects that the particular market structure of one buyer and multiple possible sellers per request has on the problem.

A straightforward approach in this situation would be that the buyer runs a reverse auction among the sellers and outsources the request to the seller bidding the lowest cost. However, this would not fulfill the EF condition. The main difference of the current setting to a standard procurement auction is that the buyer can also perform the request herself. Thus, the buyer has a reservation price, above which the auction will not lead to a trade.

EF of the resulting allocation requires that the buyer performs the request herself if and only if the buyer's own costs are lower than the costs of any seller. However, a rational buyer would keep the request to herself whenever the price that must be paid to the winning seller exceeds the buyer's own costs for performing the request. Therefore, the auction will only lead to an EF allocation if the price to be paid to the winning seller equals the lowest cost of any seller. This would imply a first price auction. However, in a first price auction, sellers have an incentive to submit bids that are higher than their true costs. If the buyer runs a second price auction 62, the sellers will bid their true costs, but the buyer has to pay a price that is higher than the lowest cost. According to the revenue equivalence theorem [42,66], the buyer will have to pay that same price (which exceeds the minimum seller's costs) in any type of auction and therefore no auction run by the buyer will be EF.

An EF allocation can be achieved by the following mechanism: The buyer informs an independent auctioneer about her reservation price for a request. The auctioneer then runs a second price auction for the request. If the lowest bid in the auction is below the stated reservation price, the transfer is made and the buyer pays the amount of the lowest bid to the auctioneer, who then pays the amount of the second lowest bid to the winning seller. If the lowest bid in the auction exceeds the buyer's stated reservation price, no transfer is made.

This mechanism is IC for both the buyer and the sellers. IC for sellers is ensured by running a second price auction among them 62. It is also IC for the buyer. This can be seen as follows: from the buyer's perspective, the mechanism basically corresponds to the BDM procedure [10] that was developed to elicit utility functions and that is also widely used in consumer research to obtain a customer's true willingness to pay for a product. In the BDM procedure, a buyer states a price b and then a random value r is drawn. If r>b, no deal is made, if r≤b, the buyer can obtain the product for the price of r. In the present setting, the random value is replaced by the lowest bid from the auction.

**Figure 2:**
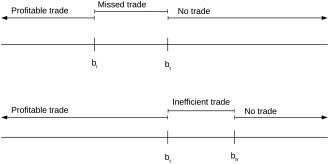
IC of a BDM mechanism

That this mechanism is IC can be shown by a simple dominance argument, as illustrated in Figure 2. Consider first the case that the buyer states a reservation price $b_{l}$ that is lower than her true costs $b_{t}$. In case the lowest bid r is between $b_{l}$ and $b_{t}$, the buyer has to perform the request herself (at her true costs) although she could outsource it for a lower price, leading to a loss of $b_{t}-r$. If the lowest price is outside that interval, deviating will lead to the same result as stating the true costs, so stating the true costs dominates specifying a lower reservation price. Likewise, if the buyer specifies a reservation price $b_{h}$ that is above the true costs, and $b_{t}\leq r\leq b_{h}$, the buyer would have to outsource the request although she could perform it cheaper herself, leading to a loss of $r-b_{t}$. Thus, stating the true costs also dominates overstating the reservation price. It is worth noting that the same argument can be used to show that in a second price auction, it is optimal to bid the true costs 8, 13. Here the second bid takes the role of the random value in the BDM procedure, but otherwise the argument remains exactly the same.

Since the mechanism allocates the request to the buyer only if its costs are lower than that of the cheapest seller, it will lead to an EF allocation. Obviously, the mechanism does not fulfill BB, since the auctioneer has to pay more to the winning seller than the amount it receives from the buyer. This is not surprising, since it can be shown in general that in a bilateral exchange 46 and a double side auction that involves buyers and sellers 67, no mechanism exists that fulfills both IC and EF and does not require external subsidies.

BB can be restored by charging potential buyers a participation fee to use the mechanism, which is equal to the expected loss of the auctioneer per transaction. This participation fee does not have incentive effects if it is not influenced by the buyer's reservation price (which is the case, since the amount depends only on bids made by the sellers), and if buyers have to decide about participating in the mechanism and thus commit to paying the participation fee before they know the request they are going to trade (and thus their own costs for the request). The second condition is necessary to avoid adverse selection effects, which would cause low‐cost buyers to abstain from participating. Practically, the exchange mechanism could be set up for multiple time periods, and participants (who might in future have the role of buyers for some requests and sellers for other requests) commit to long term membership and payment.

An illustrative example: to analyze whether such a mechanism is BB and IR, we consider a simplified setting with N≥3 participants and assume that true costs of all participants (one buyer and N−1 sellers) are uniformly distributed in the zero one interval. Thus, the expected costs of all participants (sorted in ascending order) are 1/(N+1),2/(N+1),…,N/(N+1). The participation fee is 1/(N+1). We have to distinguish three cases:

1. The buyer has the lowest cost 1/(N+1) among all participants. In that case, no transfer takes place. Since the buyer has paid the participation fee, the buyer's loss is 1/(N+1). There is no payment by the auctioneer, who nevertheless received the participation fee.
2. The buyer has the second lowest cost of 2/(N+1). The request is allocated to the seller with lowest cost, who receives the second lowest bid 3/(N+1) (note that the buyer's reservation price is not considered a bid). The buyer pays the participation fee 1/(N+1) and the lowest bid, which is also 1/(N+1). Total payment by the buyer equals the buyer's own cost, so the buyer makes neither a profit nor a loss. The difference between the price paid by the buyer and the price received by the seller is 2/(N+1). The participation fee covers 1/(N+1) of that difference, so the auctioneer in that case has a deficit of 1/(N+1).
3. The buyer's costs are m/(N+1), where m>2. The request is awarded to the lowest cost seller, who receives the second lowest bid 2/(N+1). The buyer pays 1/(N+1) for the lowest bid plus the participation fee and thus makes a profit of m/(N+1)−2/(N+1). The difference between the price paid by the buyer and the price received by the seller is 1/(N+1), which is covered by the participation fee. The auctioneer thus makes neither a profit nor a loss in that case.

Since the probability of the first two cases is the same, the auctioneer's profit in the first case, and the loss in the second case cancel out, leading to an expected profit of zero for the auctioneer.

The probability that the buyer is ranked at any position among the N participants is 1/N, therefore the expected profit of the buyer is

(1) $$\frac{1}{N}\left[\frac{-1}{N+1}+\sum_{m=3}^{N}\frac{m-2}{N+1}\right]=\frac{(N-2)(N-1)-2}{2N(N+1)}=\frac{N-3}{2(N+1)}$$

This value is zero for three participants, and strictly positive if there are more than three participants, so ex ante it is rational for the buyer to participate in the exchange if there are at least three participants (including herself). However, the buyer might incur a loss ex post. In case a seller is awarded the request, the seller also makes a profit of 2/(N+1) in case 2 and of 1/(N+1) in case 3. Hence, it is also rational for sellers to participate. As could be expected, the buyer's expected profit increases in the number of participants, while the seller's expected profit decreases. All these considerations refer to expected values, in a single realization, losses of both the auctioneer and the buyer are possible.

Although this model considers only a very simple situation, it already highlights the relationship between the conditions IC and EF. IC can be achieved if a request is assigned to a carrier whenever the price that this carrier has to pay as buyer (or receives as seller) is lower (higher) than the carrier's stated costs. EF requires that a request is assigned to a carrier whenever that carrier's true costs are lower than the cheapest alternative assignment. Since IC already means that the stated costs (or the bid in case of a seller) are identical to the true costs, the two requirements can be reconciled if the price equals the costs of the best alternative, that is, the opportunity costs of assigning a request to the carrier in question. This general principle is also relevant in the analysis of more complex settings in the following sections.

## 4 ONE SIDED BUNDLE EXCHANGE

In this section, we analyze a model that takes into account synergies between requests only on one side of the market, the sellers who offer to execute requests. We therefore assume that buyers are still able to specify a unique value which they are willing to pay to another carrier for taking over the request, and that this price equals the “true” costs of that carrier for performing the request herself.

In the presence of interdependencies between requests, the concept of “true costs” of a single request becomes questionable; the actual (marginal) costs of performing or not performing a request will likely depend on the set of other requests of a carrier. We admit that it is not realistic to assume that such interdependencies exist only on the side of the sellers, but not for the buyers. Yet, similar settings are commonly studied in literature 12, 22, 26, 68. We will use this setting mainly as a didactic example to show how a combinatorial auction can be used to design a mechanism that is both IC for sellers and EF, and the specific characteristics of the problem needed to achieve these properties.

### 4.1 The winner determination problem

We consider a set of K requests indexed by k∈{1,…,K} and N carriers m∈{1,…,N}. For the carrier who initially holds the request (the buyer), executing request k incurs costs $c_{k}$. Potential sellers (carriers willing to execute requests) bid on bundles. Bid i specifies a bundle (i.e., a set of requests) $T_{i}$ that the carrier is willing to execute, and a price $b_{i}$ that the carrier asks for to execute those requests. For the following analysis, it is not relevant whether bundles are created by the auctioneer (as proposed, e.g., by Ref. 26) or whether sellers themselves are allowed to propose a bundle and bid on it. Thus, we do not distinguish whether multiple bids (from different sellers) exist for the same bundle or not. Furthermore, we denote by

(2) $$\bar{T_{k}}=\{i:k\in T_{i}\}$$

the set of all bids that contain request k and by $B_{m}$ the set of all bids made by seller m. $B=\underset{m}{\cup }B_{m}$ is the set of all bids made.

For winner determination, the following mixed‐integer optimization model is solved. We define binary variables $x_{i}$ to indicate whether bid i is accepted ($x_{i}=1$) or not ($x_{i}=0$). The objective is to maximize total savings, that is,

(3) $$Z^{*}=\sum_{i}\left(\sum_{k\in T_{i}}c_{k}-b_{i}\right)x_{i}$$

Savings obtained from accepting one bid are determined as the difference between the original costs of all requests contained in the bid to the buyers, minus the price charged by the seller. Note that this will maximize total value creation only if bids $b_{i}$ correspond to the true costs of the sellers, that is, if IC for sellers is achieved.

Since each request can be transferred only once, it can be contained in at most one winning bid:

(4) $$\sum_{i\in \bar{T_{k}}}x_{i}\leq 1\;\forall k$$

Note that similarly to 12 and in contrast to other models found in literature 26, we do not require that each request is actually traded, it can simply remain with the buyer.

### 4.2 Computation of the second price

The true marginal costs of a seller for performing a bundle can only be meaningfully defined if each seller is allocated at most one bundle. Otherwise, the costs of performing requests in one bundle might depend on requests contained in other bundles that are also assigned to the seller. In particular, total costs could be underestimated if there are negative synergies between two requests, and these requests are acquired as parts of different bundles, so that their interaction is not taken into account. Therefore, we add the constraint that only one bid from each seller is accepted:

(5) $$\sum_{i\in B_{m}}x_{i}\leq 1\;\forall m$$

Finally, $x_{i}$ are defined as binary variables

(6) $$x_{i}\in \{0,1\}.$$

Combining all constraints, the overall model for winner determination in this setting is therefore

(7) $$\begin{array}{l} Z^{*}=\max\sum_{i\in B}\left(\sum_{k\in T_{i}}c_{k}-b_{i}\right)x_{i}\\ s.t.\\ \sum_{i\in \bar{T_{k}}}x_{i}\leq 1\;\forall k\\ \sum_{i\in B_{m}}x_{i}\leq 1\;\forall m\\ x_{i}\in \{0,1\}. \end{array}$$

We denote the optimal solution of model (7) by $X^{*}=(x_{1}^{*},x_{2}^{*},\ldots ,x_{n}^{*})$ and the corresponding objective value by $Z^{*}$. Note that in this model, the trivial solution $x_{i}=0\;\forall i$ is always feasible. Therefore, $Z^{*}$ is non‐negative. We now have to determine which price should be paid to the winning bidders in order to ensure IC. Obviously, paying the value of the bid allows bidders to determine the price being paid and creates incentives to state a price that is higher than the seller's true marginal costs. We therefore have to determine the price differently, based on the marginal costs to the system.

### 4.3 The VCG mechanism

An IC pricing mechanisms that induces reporting the true costs is the Vickrey‐Clarke‐Groves (VCG) mechanism, which is based on the concept of second price auctions developed by Vickrey 62 and later extended by Clarke and Wright 16 and Groves 32. This mechanism has been widely discussed in the literature (e.g.,^8^ and 13]

For every bidder m, denote by $Z_{m}^{0}$ the optimal value of the objective function (3) obtained from solving model (7) augmented by the constraint

(8) $$\sum_{j\in B_{m}}x_{j}=0$$

that is, where all bids from bidder m are excluded from consideration. Payments in the VCG mechanism are the difference between the second best solution $Z_{m}^{0}$ and the sum of the bids of all other winning bidders (except bidder m) in the first best solution. Note that in the present model, bids enter the objective function with a negative sign. Thus, the payment to sellers is

(9) $$p_{m}=b_{j(m)}+Z^{*}-Z_{m}^{0}$$

where $j(m)\in B_{m}\cap \{k:x_{k}^{*}=1\}$ is the index of the bid from bidder m accepted in $X^{*}$. For simplicity, we will from now on denote this bid as $b_{j(m)}$, that is, the bid of bidder m that is accepted in the first best solution.

Note that although $b_{j(m)}$ appears on the right‐hand side of (9), $p_{m}$ does not depend on $b_{j(m)}$. If $b_{j(m)}$ is a winning bid, then increasing $b_{j(m)}$ by some amount Δ will decrease $Z^{*}$ by the same amount, since the objective coefficient of the corresponding variable $x_{i}$ is decreased by Δ. Due to (8), bid j(m) is not accepted in the second best solution; therefore changing $b_{j(m)}$ will leave $Z_{m}^{0}$ unchanged. As the focal bidder is excluded from the model when calculating $Z_{m}^{0}$, he or she has no possibility to influence the payment.

Obviously, $p_{m}>b_{j(m)}$ holds iff $Z^{*}-Z_{m}^{0}>0$, which is the case if the bid is accepted in the optimum and vice versa, the bid is rejected if $p_{m}<b_{j(m)}$. Thus the dominance argument holds and this payment is IC. The solution maximizes value creation and therefore the mechanism is also EF. Furthermore, it is IR for sellers to participate, since the payment received for a winning bid is $p_{m}\geq b_{j(m)}$, and due to IC, $b_{j(m)}$ equals the costs of the seller, that is, the seller is never worse off by participating compared to abstaining from the auction.

The mechanism generates a non‐negative surplus for the auctioneer. Let i=j(m) be the winning bid of bidder m. Since all constraints in the model are of less or equal type, and all coefficients are non‐negative, $X_{\bar{i}}=(x_{1}^{*},x_{2}^{*},\ldots ,x_{i-1}^{*},x_{i}=0,x_{i+1}^{*},\ldots ,x_{n}^{*})$ must also be a feasible solution and therefore provides a lower bound on the optimal objective value $Z_{m}^{0}$. The objective values of $X^{*}$ and $X_{\bar{i}}$ differ exactly by the objective coefficient of $x_{i}$, and we obtain

(10) $$Z^{*}-Z_{m}^{0}\leq \sum_{k\in T_{i}}c_{k}-b_{i}$$

or

(11) $$b_{i}+Z^{*}-Z_{m}^{0}\leq \sum_{k\in T_{i}}c_{k}.$$

The total payment received from all the buyers for the requests contained in bid i therefore covers the payment made to bidder m, who is the seller of bid i. As we are not concerned here with incentive effects for buyers, the surplus could be distributed to buyers in any arbitrary way to achieve a balanced budget of the auctioneer.

## 5 DOUBLE‐SIDE BUNDLE EXCHANGE

### 5.1 The winner determination for double‐side bundle exchange

Now we consider possible extensions of the model to incorporate synergies between requests also on the buyer side. To provide a meaningful interpretation of “marginal costs” of a bid, the bid has to include all changes to that carrier's set of requests. We therefore define a bid i as a triple $\left(b_{i},S_{i},T_{i}\right)$ consisting of

b_i_ The price that the carrier is willing to pay ($b_{i}>0$) or that the carrier demands to be paid ($b_{i}<0$) if the bid is accepted. Note that the sign of $b_{i}$ is now the opposite as in the previous model, where $b_{i}>0$ indicated a payment to sellers.
S_i_ A set of requests that the carrier is willing to pass on to other carriers (outsourced requests).
T_i_ A set of requests offered by other carriers that the carrier is willing to perform (insourced requests).

Set $T_{i}$ was already introduced in the previous section. It is now complemented by set $S_{i}$ of the requests that the carrier wants to outsource. Consequently, the price can be positive or negative, depending on whether the total change in requests leads to a decrease or increase of total costs.

Similarly, to set $\bar{T_{k}}$ defined in (2), we define a set $\bar{S_{k}}$ containing all the bids in which request k is offered to be transferred:

(12) $$\bar{S_{k}}=\{i:k\in S_{i}\}$$

Assuming again that $b_{i}$ represents the true costs caused by all the changes to a carrier's set of requests as defined in bid i, and defining binary variables $x_{i}$ indicating whether bid i is accepted, total value creation is

(13) $$\sum_{i\in B}b_{i}x_{i}$$

which is to be maximized in winner determination.

The first set of constraints ensures that the market clears, that is, that each request that is outsourced by some carrier is insourced by another carrier and vice versa:

(14) $$\sum_{i\in \bar{S_{k}}}x_{i}=\sum_{i\in \bar{T_{k}}}x_{i}\;\forall k$$

Furthermore, each request can be traded at most once:

(15) $$\sum_{i\in \bar{S_{k}}}x_{i}\leq 1\;\forall k$$

Of course, (15) could also be formulated using $\bar{T_{k}}$. To provide a correct representation of marginal costs, at most one bid is accepted from each bidder:

(16) $$\sum_{i\in B_{m}}x_{i}\leq 1\;\forall m$$

Adding integrality constraints, we obtain the following model for winner determination:

(17) $$\begin{array}{l} Z^{*}=\max\sum_{i\in B}b_{i}x_{i}\\ s.t.\\ \sum_{i\in \bar{S_{k}}}x_{i}=\sum_{i\in \bar{T_{k}}}x_{i}\;\forall k\\ \sum_{i\in \bar{S_{k}}}x_{i}\leq 1\;\forall k\\ \sum_{i\in B_{m}}x_{i}\leq 1\;\forall m\\ x_{i}\in \{0,1\} \end{array}$$

Note that in this model, the trivial solution $x_{i}=0$ is still always feasible. Hence, if the model has a nontrivial solution, value is actually created. We again denote the solution of this model by $X^{*}=(x_{1}^{*},x_{2}^{*},\ldots ,x_{n}^{*})$, and the corresponding objective value by $Z^{*}$. Excluding bidder m via the constraint

(18) $$\sum_{i\in B_{m}}x_{i}=0$$

leads to a second best solution with objective value $Z_{m}^{0}$.

Based on this second best solution, payment to bidder m in the VCG mechanism is calculated as

(19) $$p_{m}^{VCG}=Z_{m}^{0}-\sum_{i\in B\setminus B_{m}}b_{i}x_{i}^{*}=Z_{m}^{0}-(Z^{*}-b_{j(m)})$$

where we again write for short $b_{j(m)}$ to identify the winning bid made by bidder m.

### 5.2 Challenges computing a second price in a double‐side bundle exchange

In a standard auction setting like the previous model, Equation (19) has a straightforward interpretation. In the single‐side model from Section 4, for an optimal solution $X^{*}=(x_{1}^{*},x_{2}^{*},\ldots ,x_{n}^{*})$, the solution excluding one winning bid i, that is, $X_{\bar{i}}=(x_{1}^{*},x_{2}^{*},\ldots ,x_{i-1}^{*},x_{i}=0,x_{i+1}^{*},\ldots ,x_{n}^{*})$ is always feasible and provides an objective value of $Z^{*}-b_{i}$. This value is a lower bound on $Z_{m}^{0}$ and the difference between $Z_{m}^{0}$ and this lower bound is the revenue generated from the second best use of resources freed by not fulfilling any bid of bidder m, who submitted bid i.

This interpretation is no longer valid in the present model. Not accepting one bid makes all the requests that are offered for transfer in that bid unavailable. This will cause all bids for these requests also to be rejected unless the request can be provided from other bids. However, since a request can be outsourced only by the carrier who initially holds that request, dropping one carrier from the model will also remove all other bids that contain this request from consideration. Therefore, in the VCG mechanism, if the winning bid of a bidder contains a request to be outsourced (i.e., Si=∅), excluding this bidder will necessarily remove more than one bid from the optimal solution (although bids that include insourcing this request can be replaced by other bids from the same sellers that do not contain this request). This can lead to an overall change in the objective value that exceeds the objective coefficient $b_{j(m)}$ of the winning bid of bidder m.

Consider a simple example of two bidders and two requests. Bidder 1 initially “owns” request 1, and Bidder 2 owns request 2, and their costs for performing each request are as indicated in Table 1. In the example, bidder 1 would bid 5−3=2 for exchanging request 1 for request 2, and bidder 2 would bid +1 for exchanging request 2 against request 1. The optimal solution of model (17) therefore has an objective value of $Z^{*}=3$. However, a model that excludes any one of the two bidders has only the trivial solution (because the other bid also cannot be accepted). Thus, one would assign a marginal value of 3 to each bid. This is more than the true value of the exchange for any bidder. If payments are determined according to the VCG mechanism, the payment to bidder 1 is the difference of the second best solution, zero, minus the bid of the second bidder, that is, 0−1=−1 (which equals the bid of bidder 1 minus the marginal costs of 3), and the payment of bidder 2 is 0−2=−2. Thus, although both bidders actually gain from the exchange, and would be willing to pay a positive price for the exchange to take place, the VCG mechanism would prescribe that each bidder receives a payment. Such a reversal was not possible in the single‐side model from Section 4. The problem here is that the total value gain cannot be distributed between two bids that can only be accepted or rejected jointly.

**Table 1:** Example for interactions between bids of different bidders

	Bidder 1	Bidder 2
Request 1	5	1
Request 2	3	2
Bid	+2	+1
VCG payment	−1	−2

### 5.3 The Team Bidder (TB) approach

The VCG mechanism calculates a payment for a bidder, not for a bid. One can thus argue that as long as the remaining bidders are still represented in solution $X_{m}^{0}$ (although possibly with other bids than in $X^{*}$, since requests outsourced by bidder m are no longer available) $p_{m}^{VCG}$ from (19) still reflects the marginal effect of excluding bidder m. However, it is also possible that some other bidders n=m, from whom one bid is accepted in $X^{*}$, are also no longer represented in $X_{m}^{0}$. In that case, the difference between $Z^{*}$ and $Z_{m}^{0}$ reflects the marginal effect of removing several bidders, and not just one bidder. This leads to the familiar problem of team production in economics 5: If changes in output cannot be allocated to the activities of specific team members, then on the one hand incentive considerations require each team member to receive the full marginal output, which on the other hand means that incentive payments exceed total output and the system cannot be BB. This effect aggravates the need for subsidies that already exists to establish IC in a two sided exchange 46.

This is somewhat similar to the fact that marginal costs cannot be determined for a single request and only for packages of requests. This similarity suggests using a similar approach and to combine multiple bidders into a “team bidder,” denoted by TB hereforth (and their bids into a “team bid”). Following the logic of the VCG mechanisms, which requires that bidders should not able to influence the price they are paying via their bids, we elaborate on the concept of “team bidders.”

We define a TB as a set of bidders for whom a bid is accepted in the solution of model (17), but no bid is accepted if the model is augmented by constraint (18). Thus, the team bidder $V_{m}$ implied by omitting bidder m is

(20) $$V_{m}=\{n:\exists j\in B_{n}:x_{j}^{*}=1\wedge \forall j\in B_{n}:x_{j}^{m}=0\}$$

and the set of bids that are removed by excluding bidder m is

(21) $$W_{m}=\{j:j\in \underset{n\in V_{m}}{\cup }B_{n}\wedge x_{j}^{*}=1\}.$$

Analogously to (19), we can calculate the joint payment to all bidders forming a team bidder $V_{m}$ as

(22) $$r_{m}=Z_{m}^{0}-(Z^{*}-\sum_{n\in V_{m}}b_{j(n)})$$

where we again write $b_{j(n)}$ for short to identify the winning bid of bidder n in $X^{*}$.

Note that by the definition of a team bidder, $Z_{m}^{0}$ is the objective value both of a model that excludes bidder m, and of a model that excludes all bidders in $V_{m}$ (since no bid of any of the bidders in $V_{m}$ is accepted in $X^{m}$). Thus, IC of the VCG mechanism implies that specifying the sum of their true evaluations as the sum of their bids is a dominant strategy for all bidders forming a team bidder if those bidders in total pay $r_{m}$ according to (22). Therefore, in the latter case, truthful bidding is a Nash equilibrium for individual bidders: If all the other bidders in the team bidder specify their true values, then the last bidder must also bid his or her true value in order that the sum of bids equals the sum of true values.

A bidder can be part of several TB. Note that if excluding any of two bidders m and n leads to the same team bidder, then $Z_{m}^{0}=Z_{n}^{0}$ must hold. This can be shown as follows: as we have already argued, $Z_{m}^{0}$ is identical to the optimal objective value of a model that excludes all bidders in $V_{m}$, and the same holds for $Z_{n}^{0}$. Since by assumption $V_{m}=V_{n}$, these two values are the optimal objective value of the same model and thus must be identical.

Note, however, that $n\in V_{m}$ does not necessarily imply $m\in V_{n}$. Excluding a bidder $n\in V_{m}$ leads to a different model, in which other bidders than those in $V_{m}$ (or no other bidder than n) may be excluded. Therefore, it is still necessary to allocate the payment for the team bidder according to (22) to individual bidders.

Denote by $p_{m}^{TB}$ the payment each bidder (of a winning bid) has to make (or receives if $p_{i}<0$) in the TB mechanism. For each team bidder $V_{m}$, the sum of payments has to equal $r_{m}$, thus we obtain the equation

(23) $$\sum_{n\in V_{m}}p_{n}^{TB}=r_{m}$$

There is one such equation for each bid that is accepted and therefore for each team bidder (which might simply be $V_{m}=\{m\}$). This means that the number of equations corresponds to the number of variables, since only bidders for whom a bid is accepted in $X^{*}$ can be part of a team bidder. However, some of these equations can be identical (e.g., in the small two‐bid example of Table 1, forcing any of the two bids to be not accepted will lead to the same outcome and therefore $V_{1}=V_{2}=\{1,2\}$ and $r_{1}=r_{2}=0$). Thus, there are instances in which the payments are uniquely determined by the system of Equations (23), as well as instances in which there are some degrees of freedom in determining the actual payments. In these cases it is not obvious whether an IR payment scheme can be found or not. In our computational study (Section 6) we show for several LTL scenarios, that there are always instances, where no IR payment scheme can be found. This holds even if the loss of the auctioneers is not compensated by the bidders. For cases of potential IR, the auctioneer would have to develop a payment scheme, which is considered fair by all bidders.

### 5.4 Comparison of the VCG and TB approaches

Since both mechanisms, VCG and TB, result from solving model (17), the allocations they generate are EF. The VCG mechanism is IC for individual bidders, in the TB mechanism, stating true costs is a Nash equilibrium for all bidders that together form a team bidder. We therefore have to compare the two mechanisms mainly in terms of BB and IR.

The net revenue of the auctioneer is the sum of all payments received from or made to bidders. For the VCG mechanism, summing up (19) over all m gives

(24) $$\sum_{m}p_{m}^{VCG}=\sum_{m}Z_{m}^{0}-N\cdot Z^{*}+\sum_{i:x_{i}^{*}=1}b_{i}.$$

Since the last term corresponds also to $Z^{*}$, the total net payment to the auctioneer is therefore

(25) $$\sum_{m}Z_{m}^{0}-(N-1)Z^{*}$$

Each $Z_{m}^{0}\leq Z^{*}$, but since the second term contains only $(N-1)Z^{*}$, the sign cannot be determined in general and depends on the cost structure of the bids. We first consider the case of an additive cost structure:

Proposition 1 *If the cost function is additive (i.e., the cost of performing two requests is the sum of the costs of performing the two requests individually), and bidders bid for all combinations of requests, the VCG mechanism will not generate a positive net payment for the auctioneer. (We remark that in this setting, it is not really necessary to perform a combinatorial auction, each request could be auctioned off by itself.)*

Under the assumptions given, each request will be assigned to the carrier who can execute it at lowest cost. Denote by $c_{k}^{0}$ the cost of the carrier who outsources request k, by $c_{k}^{1}$ the lowest cost for this request and by $c_{k}^{2}$ the second lowest cost. The request will be outsourced if $c_{k}^{1}\leq c_{k}^{2}\leq c_{k}^{0}$. The net payment to the auctioneer is the sum of all payments of all bidders according to (24). In the bid of the bidder who outsources request k, the request is accounted for as $c_{k}^{0}$, in the bid of the lowest cost bidder for the request as $c_{k}^{1}$. Therefore, the total effect of that request on $Z^{*}$ is $c_{k}^{0}-c_{k}^{1}>0$ (If $c_{k}^{0}-c_{k}^{1}\leq 0$, the request would not be outsourced). As long as both bidders are not excluded, the request has the same contribution to $Z_{m}^{0}$. If the bidder who outsources request k is excluded, the request is no longer available and its effect on $Z_{m}^{0}$ is zero. If the lowest cost bidder is excluded, the request is allocated to the second lowest cost carrier (who could be the outsourcing carrier, then the request is not traded) and the contribution to $Z_{m}^{0}$ is $c_{k}^{0}-c_{k}^{2}$. Instead of summing over all bidders, we can also sum over all requests and therefore obtain

(26) $$\sum_{m}Z_{m}^{0}-NZ^{*}=\sum_{k}\left[\left(0+c_{k}^{0}-c_{k}^{2})-2(c_{k}^{0}-c_{k}^{1}\right)\right]=\sum_{k}\left(2c_{k}^{1}-c_{k}^{0}-c_{k}^{2}\right)$$

Since each request occurs in the winning bids of the outsourcing bidder and the lowest cost bidder, the last term in (24) is

(27) $$\sum_{m}b_{j(m)}=\sum_{k}\left(c_{k}^{0}-c_{k}^{1}\right)$$

Therefore, the sum of all payments and thus, the net revenue of the auctioneer is

(28) $$\sum_{k}\left[\left(2c_{k}^{1}-c_{k}^{0}-c_{k}^{2})+(c_{k}^{0}-c_{k}^{1}\right)\right]=\sum_{k}\left(c_{k}^{1}-c_{k}^{2}\right)<0$$

In contrast to additive costs, the VCG mechanism can create a surplus for the auctioneer if the cost structure is super‐additive, that is, if there are negative synergies between requests so that the cost of performing two requests together exceeds the sum of the costs of performing each request alone.

This can be seen in the following example with two requests and three bidders. Bidder 1 wants to outsource request 1, which has costs of 89. Bidder 3 owns request 2, which has a cost of 82 to that bidder. Bidder 1 can perform request 2 for a cost of 46 when retaining request 1. If the bidder at the same time outsources request 1, the cost for performing request 2 increase to 58, because some synergy between the two requests is lost. Bidder 2 can perform request 1 alone for 71, request 2 alone for 49, and due to negative synergies both request together only for 139. Bidder 3 can perform request 1 for 81 and has no synergies between the two requests. Table 2 summarizes the bids made by the three bidders both for the case of negative synergies, and for additive costs. In that table, +1 indicates that a request is insourced, and −1 that a request is outsourced.

**Table 2:** Example for positive net payment to auctioneer in VCG

				Value	
Bidder	Bid	Req 1	Req 2	Additive	Synergies
1	1	0	1	−46	−46
1	2	−1	0	89	89
1	3	−1	1	43	31
2	4	0	1	−49	−49
2	5	1	0	−71	−71
2	6	1	1	−120	−139
3	7	0	−1	82	82
3	8	1	0	−81	−81
3	9	1	−1	1	1

It is easy to verify that the optimal solution for both cost structures consists in accepting bids 3, 5, and 7. Hence, request 1 is outsourced to bidder 2, who can perform it for a cost of 71, and request 2 is outsourced to bidder 1, who bids 43 under additive costs or 31 with synergies to outsource request 1 and insource request 2. The total objective value for additive costs is $Z^{*}=54$ and in the presence of synergies it is $Z^{*}=42$. If bidder 1 is excluded, request 2 is outsourced to bidder 2, yielding an objective value of $Z_{1}^{0}=82-49=33$ in both scenarios. If bidder 2 is excluded, the optimal solution in the additive scenario is to exchange both requests among bidders 1 and 3, resulting in an objective value of 44. In the scenario with synergies, only request 2 is outsourced to bidder 1 for an objective value of $Z_{2}^{0}=82-46=36$. Note that if bidder 1 would at the same time outsource request 1 to bidder 3, the total objective value in that scenario would only be 31+1=32. Finally, if bidder 3 is excluded, request 1 is outsourced to bidder 2, yielding an objective value $Z_{3}^{0}=89-71=18$ and request 2 is not available, this again holds for both scenarios. Payments to the three bidders in the additive scenario are therefore $p_{1}=33-54+43=22$, $p_{2}=44-54-71=-81$, and $p_{3}=18-54+82=46$. The auctioneer thus receives 22+46=68 from bidders 1 and 2 and pays 81 to bidder 2, resulting in a deficit of −13. In contrast, in the scenario with synergies, payments to the tree bidders are $p_{1}=33-42+31=22$, $p_{2}=36-42-71=-77$, and $p_{3}=18-42+82=58$ and the auctioneer thus has a surplus of +3.

The example also shows why a surplus can only occur if there are negative synergies between requests. Negative synergies of sellers decrease the objective values in comparison to an additive cost structure, and negative synergies of buyers increase them. This will affect both the first best and the second best solutions. Since a request can be outsourced only by one carrier, synergy effects of buyers present in the second best solution will be the same as in the first best solution (if the buyer is not excluded) or will be irrelevant (if the buyer is excluded). Thus, we only have to consider synergy effects of sellers. Since in the second best solutions, some requests are no longer available, the decrease in second best solutions will be at most as large as in the first best solution. This is illustrated in $Z_{2}^{0}$ in the above example, where both solutions decrease by the same amount since the same negative synergies affect both solutions. Other second best solutions are affected less or not at all (as the other two second best solutions in our example). Thus, the difference between first and second best solutions decreases and payments made by bidders in the presence of negative synergies will be greater or equal payments under an additive cost structure (if costs for each single request are the same). Since we have shown that under an additive cost structure, the VCG mechanism yields a nonpositive net revenue of the auctioneer, only negative synergies can make the revenue of the auctioneer positive.

It is therefore likely that the VCG mechanism generates a deficit for the auctioneer, which can be compared to the possible deficit in the TB mechanism:

Proposition 2 *The VCG mechanism leads to a net revenue of the auctioneer that is less than or equal to the net revenue of the TB mechanism*.

If a bidder is not part of any team bidder, that bidder will pay (or receive) the same amount under both mechanisms. The total payment of all bidders in a team bid is $r_{m}$, the total payment to the same bidders under VCG is

(29) ∑n∈VmZn0+∑n∈Vmbj(n)−|Vm|Z∗=Zm0+∑n∈Vmbj(n)−Z∗+∑n∈Vm,n=m(Zm0−Z∗)=rm+∑n∈Vm,n=m(Zm0−Z∗)

where we denote the winning bid of bidder n by $b_{j(n)}$ for short. Since $Z_{m}^{0}\leq Z^{*}$, the last term is not positive.

The TB mechanism therefore alleviates the problem of the VCG mechanism that it creates a low and for many cases negative revenue for the auctioneer 8, 62. However, it should also be noted that since the re‐allocation creates value, it is in both mechanisms possible to compensate the auctioneer for the loss it incurs.

### 5.5 Establishing BB through a participation fee

Proposition 3 *Any deficit of the auctioneer created by any payment scheme can be covered by the profits of the bidders*.

The deficit of the auctioneer is $\sum_{m}p_{m}$. Each bidder's profit is $b_{j(m)}-p_{m}$, and if the exchange creates value and consequently $\sum_{m}b_{j(m)}>0$, obviously

(30) $$\sum_{m}\left(b_{j(m)}-p_{m}\right)>-\sum_{m}p_{m}$$

This is basically an application of the value maximization theorem [43] which states that under the assumption of absence of wealth effects an allocation is EF if and only if it maximizes value. For each allocation that is not value maximizing, it is possible to construct a redistribution scheme so that the redistributed payoffs of the value maximizing allocation strictly dominate the nonvalue‐maximizing allocation. Absence of wealth effects basically means that utility functions of all actors are additively separable and linear in money (or whatever good is used for redistribution). Since we deal with monetary profits, this assumption is fulfilled in our case, and therefore, once value maximization is obtained, it is possible to redistribute profit to the auctioneer to make its net payoff non‐negative.

This means that similarly to the mechanism discussed in Section 3, it is possible to require payment of a fixed participation fee from carriers who want to participate in the exchange. This fee covers the expected deficit of the auctioneer and participation in the mechanism is ex ante rational for bidders (although ex post, the profit of a bidder might be less than the participation fee).

Without such a participation fee, the VCG mechanism is always IR for bidders, that is, $b_{j(m)}-p_{m}>0$. We can rewrite (19) as

(31) $$p_{m}^{VCG}=b_{j(m)}-(Z^{*}-Z_{m}^{0})\leq b_{j(m)}$$

The same holds at the aggregate level for each team bidder, but it can happen that the allocation of the payment of the team bidder to individual bidders leads to a situation in which payment exceeds a bidder's bid. IR of the TB mechanism only holds in special cases:

Proposition 4 *The payment scheme of the TB mechanism is IR in problems in which there are two winning bids*.

If the optimal solution to the winner determination problem contains two bids (from two bidders), these bids must complement each other, that is, all requests outsourced in one bid must be insourced in the other bid and vice versa. Without loss of generality, we thus can represent the problem as containing three requests.

If there are two winning bids, which we denote by 1 and 2, the following combinations are possible:

$W_{1}=\{1\},W_{2}=\{2\}$, that is, each bid alone is a team bid and can be realized without the other winning bid, thus each bidder is a team bidder;
$W_{1}=\{1\},W_{2}=\{1,2\}$ or $W_{1}=\{1,2\},W_{2}=\{2\}$, that is, one of the two bids can be realized without the other one, but the other bid cannot;
$W_{1}=W_{2}=\{1,2\}$, that is, both bids can only be realized together.

Consider first case 3, where there is only one equation (23):

(32) $$p_{1}+p_{2}=r_{1}=r_{2}=b_{1}+b_{2}-(Z^{*}-Z_{j}^{0})$$

Since both bidders are part of the same team bidder, dropping any of them will lead to the same new optimal objective value, which we denote by $Z_{j}^{0}$. Since bids 1 and 2 are the two winning bids in the original problem, we also have

(33) $$Z^{*}=b_{1}+b_{2},$$

and therefore

(34) $$p_{1}+p_{2}=Z_{j}^{0}<Z^{*}.$$

by optimality of the original solution. Therefore, the sum of payments by both carriers is less than the sum of their bids, and it is thus possible to construct a payment scheme in which each carrier pays less or receives more than its original bid (which, by IC, is equal to the carrier's costs).

Now consider case 2. Here, one of the two winning bids can be replaced by some other combination of bids. The structure of bids is given in Table 3.

Note that in general, there can be more than one additional bid and also additional requests, and there can also be several additional bids that are in total needed to provide all the requests insourced by bid 1. In the following proof, we will only use the net effect of these additional bids, which is represented by $b_{4}$ above.

The optimal solution of the original problem is to accept bids 1 and 2 with an objective value of $Z^{*}=b_{1}+b_{2}$. If bidder 1 is excluded, the optimal solution is to accept bids 3 and 4 with an objective value $b_{3}+b_{4}$ or to accept no bid. The latter case would correspond to case 3 above, so we here must assume that $b_{3}+b_{4}>0$. Thus, bidder 1 is a team bidder by itself and $V_{1}=\{1\}$. Therefore, the payment of that bidder is $p_{1}=r_{1}$, which is given by

(35) $$p_{1}=r_{1}=Z_{1}^{0}-Z^{*}+b_{1}=(b_{3}+b_{4})-(b_{1}+b_{2})+b_{1}=b_{3}+b_{4}-b_{2}$$

From optimality of the original solution, we know that

(36) $$b_{1}+b_{2}\geq b_{3}+b_{4}$$

and thus,

(37) $$b_{1}\geq b_{3}+b_{4}-b_{2}=p_{1}.$$

So, bidder 1 has to pay at most his or her original bid.

Dropping bidder 2, who is the only one willing to insource request 2, leads to the trivial solution of accepting no bid, so we have $V_{2}=\{1,2\}$ and

(38) $$r_{2}=Z_{j}^{0}-Z_{j}^{1}+(b_{1}+b_{2})=0-(b_{1}+b_{2})+(b_{1}+b_{2})=0.$$

Since $p_{1}+p_{2}=r_{2}=0$, we obtain $p_{2}=-p_{1}=-b_{3}-b_{4}+b_{2}$. From optimality of the second best solution when dropping bid 1, we know that

(39) $$Z_{1}^{0}=b_{3}+b_{4}\geq 0,$$

and therefore,

(40) $$p_{2}=b_{2}-b_{3}-b_{4}\leq b_{2}.$$

Therefore, also bidder 2 pays at most the original bid.

The same argument that we used above for bidder 1 can, mutatis mutandis, also be used in case 1, so also in that case both bidders will pay at most their original bids.

**Table 3:** Structure of bids in case 2

Bidder	Bid	Req.^1^	Req.^2^	$b_{i}$
1	1	+1	−1	$b_{1}$
2	2	−1	+1	$b_{2}$
2	3	−1	0	$b_{3}$
3	4	1	0	$b_{4}$

**Figure 3:**
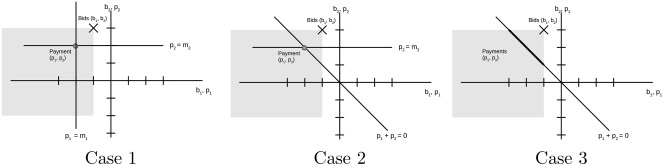
IR of payment schemes for two winning bids

Figure 3 shows the three cases graphically. In the first case, both bids can be performed independently, thus we have two equations that each define the payment for one bidder. Since we have shown that for each bidder, the payment must be less than or equal to that bidder's bid, the intersection of the two lines dominates the original bids. In case 2, there is one equation of the form $p_{j}=r_{j}$, which determines that bidder's payment, and the condition that payments sum up to zero. Again, we have shown that the intersection of the two lines dominates the original payments. In the third case, we only have the condition that $p_{1}+p_{2}=0$, so the solution is not unique, but there are points on that line that dominate the original bids.

Case 3 above points to a first potential problem. There are solutions that fulfill IR, but there are also solutions, which violate it (i.e., in which one carrier has to pay more than his or her bid). Therefore, one has to select one of the possible solutions that fulfills this condition. It would be tempting to use an optimization model that e.g., maximizes the smallest surplus among bidders, that is, that uses the objective function

(41) $$\max\underset{m}{\min}b_{j(m)}-p_{m}$$

However, such a model would lead to a violation of IC, since then the price a bidder has to pay depends on that bidder's bid, creating an incentive to deviate from the true value.

Furthermore, if there are more than two winning bids, IR can no longer be guaranteed. Consider the example shown in Table 4, which contains 2 requests and 3 bidders. Bidder 1 just wants to outsource a request (request 1), bidder 2 is willing to accept request 2, and bidder 3 can both insource request 1 and outsource request 2.

**Table 4:** Example in which TB violates IR

Bidder	Bid	Req. 1	Req. 2	Value
1	1	−1	0	62
2	2	0	1	−1
3	3	0	−1	50
3	4	1	0	−31
3	5	1	−1	20

The optimal solution is to accept bids 1, 2, and 5, that is, to transfer both requests, which gives an objective value of 81. If bidder 1 is excluded, bidder 3 can still outsource request 2 to bidder 2, which gives an objective value of 49. If bidder 2 is excluded, request 1 can be outsourced from bidder 1 to bidder 3 resulting in an objective value of 31, and if bidder 3 is excluded, no trade is possible. The VCG payments in this example are $p_{1}^{VCG}=49-81+62=30$, $p_{2}^{VCG}=31-81-1=-51$, and $p_{3}^{VCG}=0-81+20=-61$, leading to a total deficit of the auctioneer of 82. Note that although bidder 3 creates value only jointly with bidders 1 and 2, all the value creation (minus the own bid) is allocated to that bidder.

Since bidders 1 and 2 are team bidders of their own, the corresponding equations contain only their prices and the right‐hand sides correspond to their VGC payments, so these two bidders pay the same amount as in the VCG mechanism. However, for the third bidder, the right‐hand side of the equation is zero (0−81+81), thus payment of this bidder is −30+51=21 which is more than the amount of that bidder's winning bid (20).

This example also shows another property of the TB mechanisms.

Proposition 5 *Whenever all bidders together form a team bidder (i.e., for some bidder* m, $|V_{m}|=N$ *), the net payoff to the auctioneer in the TB mechanism is zero*.

If all bidders together form a team bidder, that means that no bid is accepted in the second best solution for one bidder, and thus $Z_{m}^{0}=0$ for that bidder. The corresponding right‐hand side is therefore $Z^{*}-\sum_{m\in V_{m}}b_{j(m)}$ and since all bidders are part of the team bidder, $Z^{*}=\sum_{m\in V_{m}}b_{j(m)}$ and therefore, the right‐hand side is zero. Since the team bidder encompasses all bidders, the left‐hand side is the sum of payments to all bidders, which is identical to the net payoff of the auctioneer, which therefore must be zero.

To summarize our results so far, the TB mechanism may violate IR ex ante, but leads to a lower deficit of the auctioneer. On the other hand, the VGC mechanism guarantees IR ex ante, but leads to a larger deficit of the auctioneer. Thus, if the auctioneer's deficit is compensated by a fixed participation fee, it is a priori not clear which mechanism will more often lead to IR ex post. This is the main trade‐off between the two mechanisms.

## 6 COMPUTATIONAL STUDY

We numerically analyze the mechanism described in Section 5 by applying it within the auction‐based transportation exchange framework proposed by Gansterer and Hartl 27. We focus on carriers having LTL pickup and delivery requests, that is, multiple pickups or deliveries occur along the route of a vehicle. A carrier starts at the depot, visits a given set of paired pickup and delivery nodes and returns to the depot again. Precedence constraints have to be observed, that is, the pickup has to be performed before the corresponding delivery. The objective is to minimize total travel time. We assume that the carriers are willing to exchange their requests with their competitors, aiming for an increase in efficiency and sustainability. A traded item, that is, a request, is a transportation order going from a pickup to its corresponding delivery node. We note, however, that all the considerations of the previous sections also hold for unpaired pickup and delivery problems or problems with only pickups or only deliveries.

A mathematical model of the optimization problem faced by each carrier is given in 26. It belongs to the class of vehicle routing problems with precedence constraints, and is referred to in 8 as the single‐vehicle variant of the vehicle routing problem with pickups and deliveries (SPDP) and in 11 as the one‐to‐one pickup and delivery problem.

Each carrier has to select a set of transportation requests to submit to the request pool. Gansterer and Hartl 26 show that these should be requests that are relatively close to each other. If carriers submit unattractive requests, like outliers being far away from other locations, the total collaboration profits are significantly decreased. The distance between two requests is taken to be the distance between their origins (pickup locations) plus the distance between their destinations (delivery locations).

Transportation requests in the request pool are packaged into bundles by the auctioneer. Without any information on the transportation requests of a carrier, the auctioneer builds these bundles based on the geographic information associated with the transportation requests in the request pool. Gansterer et al. 30 show that an auctioneer can generate effective bundles using a genetic algorithm in which a bundle is evaluated based on (i) its isolation, that is, the distance to other bundles, (ii) its density, that is, the ratio of the area covered by the requests and the number of requests, and (iii) the length of the tour required to serve the requests.

The carriers determine their bids i as a triple $\left(b_{i},S_{i},T_{i}\right)$, as described in Section 5. The bid value $b_{i}$ is calculated by the marginal profit of insourcing requests in $T_{i}$ and outsourcing requests in $S_{i}$. We do this in order to let the bid reflect all changes to that carrier's set of requests. Marginal profits are determined using a simple insertion heuristic 30 (Note that in 30 we have shown that the outcome of the auction mechanism is not significantly influenced if a correct computation of the marginal profit is replaced by an approximate one by a simple insertion algorithm.). We impose that a carrier wins at most one bundle so as to guarantee that the acquired requests meet with the carrier specific capacity constraints. If all possible bundles would be offered, this condition is satisfied naturally. Because not all possible bundles are offered, we cannot guarantee that all feasible combinations are offered. But the chances of that happening are small because of the way the bundles are generated, and the computational advantages of imposing this restriction are considerable. Based on the bids, the winner determination finds the optimal assignment of bundles to carriers, where constraints (14)‐(16) have to hold.

Following 12 we use self‐created random instances. These instances consider different scenarios in terms of (i) degree of customer overlaps and (ii) distance of requests to the carriers' depots. We assume that three carriers operate in overlapping but not identical customer regions. There are three types of instances depending on the degree of customer area overlaps (O1_xx, O2_xx, O3_xx). For each instance, we generate equidistant carrier depots with a distance of 200. Requests are randomly generated within a radius of 150 (O1), 200 (O2), and 300 (O3) from their carrier's depot. Each carrier initially has up to 15 requests and offers 5 of them for trading. We generate 20 instances for each scenario. All instances are publicly available (http://prolog.univie.ac.at/research/BundlingInst/Bundling_instances.zip). Experiments are coded in C++ and executed single‐threaded on an Intel Core i5–3570 3.4GHz computer.

The computational study is used to compare VCG against the TB mechanism, applied to three different settings of carrier collaborations. Since both mechanism are IC and EF (see Section 5), we focus on the properties of BB and IR. While VCG is IR ex ante, this property does not hold for the proposed TB mechanism. Thus, we first quantify how frequently IR is violated. None of the mechanisms is BB, and VCG will per definition lead to a higher loss for the auctioneer. As it is discussed in Section 5, this loss can be compensated by a fee paid by the carriers. We assume that the auctioneer's loss is distributed equally to the carriers. Obviously, such a fee might lead to payments that violate IR. We therefore evaluate, whether IR holds ex post, if carriers are charged with a participation fee.

To answer the first question, we generate constraints (22) and (23) for each team bidder, and check whether the system of equations has a unique solution for $p_{1}$, $p_{2}$, and $p_{3}$. If this is not the case, we check the system for possible IR solutions by solving the following optimization model:

(42) $$\begin{array}{l} \max z\\ s.t.\\ \sum_{j\in W_{i}}p_{j}s_{ij}=r_{i}\;\forall i\in W\\ p_{j}+z\leq b_{j}\;\forall j\in W\\ -\infty <p_{i},z<\infty \;\forall i\in W, \end{array}$$

where $s_{ij}$ indicates whether bidder j is part of team bid i (1) or not (0). If z becomes negative, we know that no IR solution can be reached.

The auctioneer's loss can be calculated by summing up values $p_{j}$ in both mechanisms. If the sum is negative, the auctioneer makes a loss. We summarize the results in Table 5, where IR gives the percentage of instances where IR is violated for at least 1 carrier. In column AL we provide the average auctioneer's loss in % of the total initial profit, and IR ex post gives the percentage of instances where IR is violated for at least 1 carrier if the AL is distributed equally.

**Table 5:** Analysis on IR of VCG and the TB mechanism applied to instances O1, O2, O3

	VCG			Team Bidder		
Instance	IR	AL	IR ex post	IR	AL	IR ex post
O1	0.0%	1.5%	52.5%	5.0%	0.4%	5.0%
O2	0.0%	4.9%	42.5%	20.0%	2.6%	35.0%
O3	0.0%	7.5%	35.0%	30.0%	2.3%	35.0%

We observe that for both mechanisms the loss of the auctioneer increases with the degree of overlaps in the customer areas. This can be explained by the fact that more overlaps offer more possibilities for profitable exchanges. In both mechanisms this leads to higher payments for the auctioneer (see Section 5). While VCG is 100% IR if carriers do not have to pay a participation fee, IR is violated if the auctioneer's loss has to be compensated. When distributing the loss equally among participants, no IR solution can be reached in 52.5% (O1), 42.5% (O2), and 35.0% (O3) of the instances. However, the participation fee has no major impact on IR of the TB mechanism. Without the participation fee, no IR solution could be reached in 5.0% (O1), 20.0% (O2), and 30.0% (O3), which is relatively close to the results with participation fee.

From these observations, we conclude that both of the proposed mechanisms come with some limitations with regard to the four desired properties. VCG is EF, and IC, but not BB, and not IR as soon as carriers have to compensate auctioneers' losses. The TB mechanism is less costly for the auctioneer, but IR cannot be guaranteed. Even if carriers do not pay for the loss of the auctioneer, there might be participants who by participating are worse‐off.

Our study shows that strategic behavior in auction‐based carrier collaborations can be avoided, but might violate IR of participants. It cannot be guaranteed that all carriers benefit from participation. Vice versa, if carriers insist on proven IR, untruthful behavior might become the dominant strategy. Thus, an appropriate mechanism for the collaborative exchange of transportation request—if such a mechanism exists—is yet to be discovered.

## 7 CONCLUSIONS

Collaboration has been one of the important trends in vehicle routing since the potential of further reducing the mileage driven by a single carrier is limited, as increasingly sophisticated optimization methods and tools are being used. By exchanging some requests between carriers, inefficiencies in carrier operations can be reduced and economic benefits for the participants can be generated. Furthermore, in doing so, environmental goals can be reached, by reducing traffic, pollution, and accidents. One of the possible mechanisms to enable carrier collaboration is to use combinatorial auctions. In combinatorial auctions, requests are not traded individually but are combined into packages (or bundles). The main reason for this is that bundles might have a different value to the partners than the sum of the individual requests. Previous literature on carrier collaboration has considered parts of such mechanisms such as bundle formation or winner determination mainly in isolation, typically also assuming truthfulness of all agents and absence of any strategic behavior. In the present article, we also consider the interdependencies that arise from bidders acting as buyers and sellers of bundles of requests at the same time, and the difficulties these interdependencies create for developing incentive compatible mechanisms.

From standard auction theory literature, we have identified desirable properties of exchange mechanisms as efficiency (EF, value creation from exchange is maximized), incentive compatibility (IC, bidding the true valuation is the optimal strategy for each agent), IR (participating should not lead to a loss for a bidder), and BB (auctioneer does not incur a loss). From the revelation principle of standard auction theory, we have concluded that IC does not restrict the possibility to find an adequate mechanism for a problem, which is why we focused on mechanisms, which satisfy IC. Also from standard auction theory, we have concluded that in double auctions, the desirable properties mentioned above cannot be fulfilled at the same time. In particular, the properties IC and EF induce that BB is violated, that is, an outside subsidy is required.

Compared to standard situations in auction theory, our situation is more complicated because we have a double auction, involving multiple buyers and multiple sellers. Even more problematic is the fact that carriers can be (and typically are) buyers and sellers at the same time. Therefore, it is impossible to compute the real valuation of a set of requests to be offered and of a set of requests to be received, independently. The insertion cost of new requests depends on which requests can be outsourced or have to be kept. As a consequence, one has to consider bundles of requests being outsourced and bundles of requests being insourced simultaneously.

Auction theory suggests that the established IC, payments of actors should not depend on their own bids (otherwise, there is a clear incentive to act strategically). This is typically done using a second price sealed bid auction mechanism (also known as Vickrey‐Clarke‐Groves mechanism, VCG). Since we want to stay with IC mechanisms, we focus on these types of auctions. However, the fact that requests are unique and only one bidder can act as a buyer aggravates the problem that this mechanism requires a subsidy. Systematic over‐estimation of the marginal effects of dropping a request may even lead to a situation in which the auctioneer would have to make payments to all participants. We proposed and investigated two exchange mechanisms to deal with the resulting deficit.

One is more closely related to the classical VCG approach, while in the other one, called “team bidder” (TB) we propose a more complicated concept for computing payments to participants. We have shown that if payments to participants are computed using standard VCG rules, and the resulting deficit of the auctioneer is then also charged to participants, this might ex post lead to violations of IR. Taking into account that interdependencies can led to a situation known as team production in economics, in which individual contributions of participants can no longer be separated, reduces the auctioneer's deficit, but could lead to violations of IR even ex ante (without contributions to cover the auctioneer's deficit).

A numerical study has complemented the above theoretical findings and has shown the following important observations

- While VCG is ex ante IR and TB is not, compensating the auctioneer for the loss leads ex post to more violations of IR by the classical VCG approach than when applying the TB mechanism.
- The more the regions of the carriers overlap (less clustered customers), the more requests are exchanged and the higher the loss of the auctioneer, and hence the required participation fee.
- The ex post violations of IR are more frequently encountered for the VCG approach than for the TB setting. However, as the overlap of the carriers' customer regions increases the difference between these two settings decreases and disappears for very high overlap.

We conclude that both mechanisms can be acceptable in practical situations. On average, all participants benefit from the exchange, since the collaboration gain cannot be negative. While some of them might incur a loss after compensating the auctioneer through a participation fee, this is not problematic if the collaboration lasts for many periods, and occasional losses are evened out by profits in other periods. In this sense, IR will be satisfied in the long run. Only if the problem is very asymmetric and some carrier makes a loss very often, also long run IR might be violated and he has an incentive to leave the collaborative mechanism.

The focus of this paper was to highlight the problems encountered when aiming for an exchange mechanism that is IC, since this is the typical point of view in auction theory. Consequently, we have investigated two alternative approaches, VCG and TB, which are both IC. One might wonder, whether IC is worth the price of having to compensate the auctioneers loss. A straightforward alternative approach, which is BB, would be to avoid such an auctioneers loss by paying only the second price to the seller. After all, the buyer also only pays the second price, and this is also current practice in consumer‐to‐consumer auctions on online market places. While such a mechanism is still IC for the buyer, it is clearly not IC for the seller who now has an incentive to charge a higher price than the true valuation for his service. While our article has focused on IC in the tradeoff between IC and BB, it might be interesting to also investigate other mechanisms, which give in on the property of IC (which in any case is a theoretical property based on the assumption of perfectly rational agents) and in contrast put more emphasis on achieving BB.

This is in line with most previous work on carrier collaboration, which while ignoring aspects of strategic behavior has (implicitly) assumed that a first price auction is used. This is clearly not IC, neither for the buyer, nor the seller. However, it is often not clear in which direction and how much bidders should deviate from the true valuation, and in the end, strategic behavior might not be such big problem. Investigating this aspect might be an interesting topic for experimental economics.

Another insight that we obtained from our study is that the balance between different desirable properties of an exchange mechanism is heavily influenced by the cost structure of involved carriers. The extent to which synergies between requests are present, and whether omitting one carrier from consideration has large repercussions on trades between other carriers significantly impacts the auctioneer's deficit and thus how much other properties have to be weakened to restore BB. Fully understanding these effects requires significant future research, both theoretically and via additional computational studies.

## APPENDIX 1 LIST OF VARIABLES

n,m∈{1,…,N}: Bidders (carriers)
i,j∈{1,…,J}: Bids
$B_{m}\subseteq \{1,\ldots ,J\}$: Set of bids made by carrier m
B={1,…,J}: Set of all bids
$V_{m}\subseteq \{1,\ldots ,N\}$: team bidder of all bidders excluded together with bidder m
$W_{m}$: Bids made by bidders in $V_{m}$
k∈R={1,…,K}: Requests
$c_{k}$: True costs of request k for outsourcing carrier
$b_{i}$: Amount (payment)of bid i
$S_{i}$: requests outsourced in bid i
$T_{i}$: requests insourced in bid i
$p_{m}^{VCG},p_{m}^{TB}$: payment of bidder m in VCG and Team bidder mechanisms
